# Comprehensive Analysis of the Synergistic Effects of Bimetallic Oxides in CoM/γ-Al_2_O_3_ (M = Cu, Fe, or Ni) Catalysts for Enhancing Toluene Combustion Efficiency

**DOI:** 10.3390/molecules30051188

**Published:** 2025-03-06

**Authors:** Yuwei Tang, Xu Yang, Qinglong Zhang, Dongmei Lv, Shufeng Zuo, Jing Li

**Affiliations:** 1 Shandong Provincial Key Laboratory of Chemical Energy Storage and Novel Cell Technology, College of Chemistry and Chemical Engineering, Liaocheng University, Liaocheng 252059, China; tangyuweiyibo@163.com (Y.T.); yxv2021@163.com (X.Y.); a1942213564@163.com (Q.Z.); lvdongmei@lcu.edu.cn (D.L.); 2 Zhejiang Key Laboratory of Alternative Technologies for Fine Chemicals Process, College of Chemistry and Chemical Engineering, Shaoxing University, Shaoxing 312000, China

**Keywords:** bimetallic oxides, toluene combustion, sulfur resistance, synergistic effect, acidity effect

## Abstract

Catalytic combustion is an efficient and economic technology for eliminating volatile organic compounds (VOCs) in industrial environments. This study evaluated the synergistic catalytic properties of bimetallic oxides, viz., CoM/γ-Al_2_O_3_ (M = Cu, Fe, or Ni), for improving the combustion efficiency of toluene. The CoM/γ-Al_2_O_3_ catalysts were prepared by an impregnation method and characterized by using advanced techniques. Among the bimetallic catalysts, CoCu/γ-Al_2_O_3_ exhibited the best performance. The findings revealed that owing to the strong synergistic interaction between Cu, Co, and the γ-Al_2_O_3_ support, the active species in the CoCu/γ-Al_2_O_3_ catalyst were effectively stabilized, and they significantly enhanced the redox performance and acidity of the catalyst, demonstrating superior catalytic activity and sulfur resistance. Conversely, the CoFe/γ-Al_2_O_3_ catalyst performed poorly, exhibiting a significant decline in its activity owing to sulfur poisoning. The insights from this study provide theoretical support for designing efficient, sulfur-resistant catalysts that are crucial to reducing industrial VOC emissions.

## 1. Introduction

Toluene is one of the prevalent volatile organic compounds (VOCs) in industrial production [1,2,3,4]. When the concentration of toluene in the air exceeds safe limits, it poses a significant threat to both human and environmental health. It can potentially cause neurological disorders, developmental malformations, cancer, vision and hearing loss, photochemical smog formation, and climate change [1]. The catalytic oxidation of VOCs into carbon dioxide and water is currently one of the most effective and economically viable technologies for addressing this issue [5,6,7]. Catalytic combustion employs high-efficiency catalysts, such as noble-metal catalysts. Despite their superior performance, noble-metal catalysts are expensive due to their limited availability, high cost, susceptibility to poisoning, and poor thermal stability [8,9,10]. Consequently, transition-metal catalysts have become the focus of research owing to their excellent thermal stability and environmental friendliness and the variability of their valence states, which allows for their modulation [11].

The transition-metal catalysts that are commonly applied in the catalytic oxidation of VOCs include Cu [12], Mn [13], Ce [14], Co [15,16], Fe [17], and Ni [18]. Their high catalytic performance in oxidizing VOCs is attributed to their unique valence combinations and coordination structures. Wang et al. [19] synthesized a catalyst composed of MnO_2_ supported on γ-Al_2_O_3_, which facilitated the deep catalytic combustion of toluene at 330 °C. The superior activity of this catalyst was due to the presence of Mn^3+^ and Mn^4+^ oxide species, which promoted the creation of numerous oxygen vacancies on the catalyst surface. Moreover, a larger amount of Mn^4+^ provides more active sites, favoring the deep oxidation of toluene. Niu et al. [20] utilized eggshell membranes as templates to synthesize a three-dimensional CoO_x_ catalyst network. However, the catalyst structure changed during its use in oxidation processes. Despite the presence of Co^3+^ as the active cobalt species, hydrogen spillover from Co^0^ to the catalyst surface enhanced the reducibility of eggshell membrane -CoO_x_, markedly enhancing the activity of the catalyst in toluene oxidation at lower temperatures. Jiang et al. [21] identified Co^3+^ oxide in Co_3_O_4_/γ-Al_2_O_3_ as the active site in toluene combustion. However, when γ-Al_2_O_3_ is used as the carrier, the interaction between Co and Al_2_O_3_ often results in the partial reduction of Co^3+^ to Co^2+^, which then gets fixed in the alumina lattice, resulting in the formation of inactive CoAl_2_O_4_. Consequently, this process decreases the catalytic activity of the material [22]. In addition, trace amounts of sulfur-containing VOCs can deactivate the catalyst by reacting with its active centers to form inactive sulfates, inducing a shielding effect that affects both the active components and the carrier, ultimately leading to a substantial decline in catalytic activity and lifespan [19,21]. Hence, it is imperative to enhance the low-temperature activity and sulfur resistance of active centers within transition-metal oxide-supported catalyst systems.

Compared with single-metal oxide catalysts, multi-metal oxide catalysts comprising two or more active metals often demonstrate superior catalytic activity in the oxidation of VOCs. The metal species in multi-metal oxide catalysts often exhibit synergistic effects, improving the distribution of reactive components and increasing the mobility of oxygen species. Furthermore, metal doping into multi-component systems can result in the formation of additional vacancies or defects, contributing to enhanced activity and stability of the catalyst in complex environments. Xu et al. [23] developed a series of Cu-Mn combined oxide catalysts via a hydrothermal method for catalytic toluene combustion. The most active catalyst completely eliminated toluene at temperatures below 210 °C, whereas the least active catalyst required a higher temperature of 221 °C to completely eliminate toluene. This can be attributed to the existence of two redox couples, viz., Cu^2+^/Cu^+^ and Mn^4+^/Mn^3+^ in the Cu-Mn composite oxide catalyst. These redox couples facilitate charge transfer and promote the redox reaction of Cu^+^ + Mn^4+^ ↔ Cu^2+^ + Mn^3+^, thus enhancing the redox capability of the catalyst. Barama et al. [24] investigated toluene combustion by using Ni-Mn mixed oxide catalysts synthesized through ammonium oxalate coprecipitation and citrate complexation. They confirmed that the activity of Ni-Mn materials is related to the redox and acid–base properties arising from Mn^4+^ species, which serve as Lewis acid sites, enhancing the chemical adsorption of toluene. Soltan et al. [25] loaded bimetallic Fe-Mn onto H-ZSM-5 zeolites, achieving superior catalytic efficiency and high stability in toluene oxidation at low temperatures. The good synergistic interaction between Fe and Mn in the Fe_2_-Mn/H-ZSM-5 catalyst resulted in numerous oxygen vacancies, sufficient acidic sites, and multiple types of active oxygen species, which enhanced toluene adsorption and accelerated its degradation. Xu et al. [26] developed a trimetallic oxide catalyst consisting of Co, Cu, and CeO_2_, targeting the degradation of toluene under elevated-humidity conditions. The study revealed that Cu incorporation facilitates electron movement from Co^2+^ to Co^3+^, thereby enhancing the redox capability of the catalyst. This promotes the movement and transformation of the Cu species at the nanoscale level, enabling the replacement of the lattice atoms. The dual-metal substitution boosted the capacity of CeO_2_ to generate oxygen vacancies. The results indicated that designed defects and multi-metal sites are vital to efficiently eliminating toluene from complex multi-component environments.

In this study, we synthesized a series of bimetallic oxides supported on γ-Al_2_O_3_, that is, CoM/γ-Al_2_O_3_ (M = Cu, Ni, or Fe), using an impregnation method. We then prepared sulfur-poisoned samples by thermally decomposing (NH_4_)_2_SO_4_ in situ to generate a quantitative SO_2_ source. The synergistic effects of MO_x_ and CoO_y_ on the γ-Al_2_O_3_ carrier, as well as their mutual influences, were analyzed via various techniques, including X-Ray diffraction (XRD), Fourier transform infrared (FT-IR) spectroscopy, N_2_ adsorption/desorption analysis, high-resolution transmission electron microscopy (HRTEM), energy-dispersive X-Ray spectroscopy (EDS), X-Ray photoelectron spectroscopy (XPS), and H_2_ temperature-programmed reduction (H_2_-TPR). The acidity and adsorption capacities of the CoM/γ-Al_2_O_3_ samples were evaluated based on the temperature-programmed desorption (TPD) of NH_3_, toluene, and SO_2_. Furthermore, the catalytic performance of the samples in toluene combustion and their sulfur tolerance were investigated. Finally, the structure–activity relationship of the CoM/γ-Al_2_O_3_ bimetallic oxide in the catalysis of toluene combustion was comprehensively analyzed. This study provides a theoretical foundation for the comprehensive exploration of high-performance multi-metal catalytic systems and their potential application in complex environments.

## 2. Results and Discussion

### 2.1. Synergistic Effects

In multi-component catalytic systems, interactions between the constituent metals or oxides can not only modify the surface structure and chemical properties of the catalyst but also lead to synergistic effects. These synergistic effects contribute to the improved catalytic activity and selectivity of multi-metal oxide catalysts during catalytic reactions [27].

**Figure 1:**
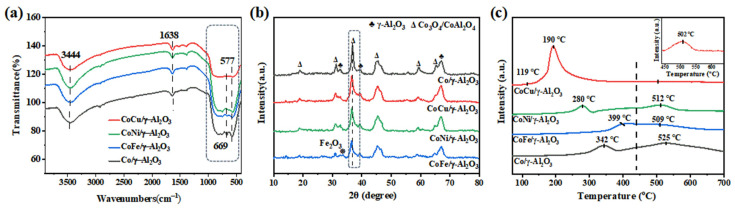
(**a**) FT-IR spectra, (**b**) XRD patterns, and (**c**) H_2_-TPR curves of the Co/γ-Al_2_O_3_ and CoM/γ-Al_2_O_3_ (M = Cu, Ni, or Fe) catalysts. * was used to mark the characteristic peak positions of Fe_2_O_3_ in XRD.

As shown in Figure 1a, our previous study demonstrated that the Co/γ-Al_2_O_3_ catalyst exhibits two distinct sets of FT-IR peaks: O-H stretching vibrational peaks (3444 and 1638 cm^−1^) attributed to H_2_O [28,29] and the Al-O and Co-O vibrational peaks (669 and 577 cm^−1^) associated with the spinel CoAl_2_O_4_ formed due the incorporation of Co into the γ-Al_2_O_3_ support [30,31,32]. The introduction of Cu, Ni, or Fe resulted in a decrease in the peak intensity of CoAl_2_O_4_, mainly because of the reduction in Co content within the catalyst. Compared with CoNi/γ-Al_2_O_3_ and CoFe/γ-Al_2_O_3_, the CoCu/γ-Al_2_O_3_ catalyst presented a notably weaker peak intensity of CoAl_2_O_4_, indicating that Cu impedes the incorporation of Co species into the γ-Al_2_O_3_ support, thereby hindering the formation of CoAl_2_O_4_ [22]. Compared with the single-metal Co/γ-Al_2_O_3_ catalyst, the bimetallic CoCu/γ-Al_2_O_3_, CoNi/γ-Al_2_O_3_, and CoFe/γ-Al_2_O_3_ catalysts exhibited decreased intensities of the main diffraction peaks of Co_3_O_4_ and CoAl_2_O_4_ spinel phases at 36.5° (Figure 1b) owing to reduced Co content. However, the overall structures of the catalysts remained unchanged. For the bimetallic catalysts, the diffraction peak intensity at 36.5° followed the order of CoCu/γ-Al_2_O_3_ > CoNi/γ-Al_2_O_3_ > CoFe/γ-Al_2_O_3_, with the strongest intensity being observed for CoCu/γ-Al_2_O_3_. FT-IR analyses revealed that the CoCu/γ-Al_2_O_3_ catalyst formed the lowest amount of CoAl_2_O_4_, indicating higher Co_3_O_4_ formation. Thus, Cu introduction enhanced the stability of Co_3_O_4_ loaded on the γ-Al_2_O_3_ support. Furthermore, neither CoCu/γ-Al_2_O_3_ nor CoNi/γ-Al_2_O_3_ exhibited diffraction peaks for individual Cu or Ni species, whereas CoFe/γ-Al_2_O_3_ showed a diffraction peak for Fe_2_O_3_ [33,34,35,36]. This result indicates that Cu and Ni species were highly dispersed on the γ-Al_2_O_3_ support, whereas the Fe species were poorly dispersed on the support surface. The H_2_-TPR curve (Figure 1c) of the CoCu/γ-Al_2_O_3_ catalyst exhibited a remarkable reduction peak at 190 °C, indicative of the reduction of strongly interacting Cu and Co oxides. The strong reduction peak suggests improved redox performance of the catalyst. At 502 °C, a weak reduction peak of CoAl_2_O_4_ was observed, consistent with the FT-IR results [22]. The reduction temperatures of Ni and Co oxides in CoNi/γ-Al_2_O_3_ were lower than those of the corresponding species in Co/γ-Al_2_O_3_, suggesting weaker interactions between Ni and Co. In the CoFe/γ-Al_2_O_3_ catalyst, the aggregation of Fe_2_O_3_ particles led to an increase in the reduction temperatures of Fe and Co oxides. In summary, the CoCu/γ-Al_2_O_3_ system exhibited strong interactions between Cu and Co, which inhibited the formation of inactive CoAl_2_O_4_. Thus, more of the active Co_3_O_4_ species were retained in the catalyst, resulting in enhanced redox performance. While the CoNi/γ-Al_2_O_3_ catalyst had weak interactions between Ni and Co, the CoFe/γ-Al_2_O_3_ catalyst had poor interactions between Fe and Co, which impeded the dispersion of Fe species and consequently diminished its redox capacity.

**Figure 2:**
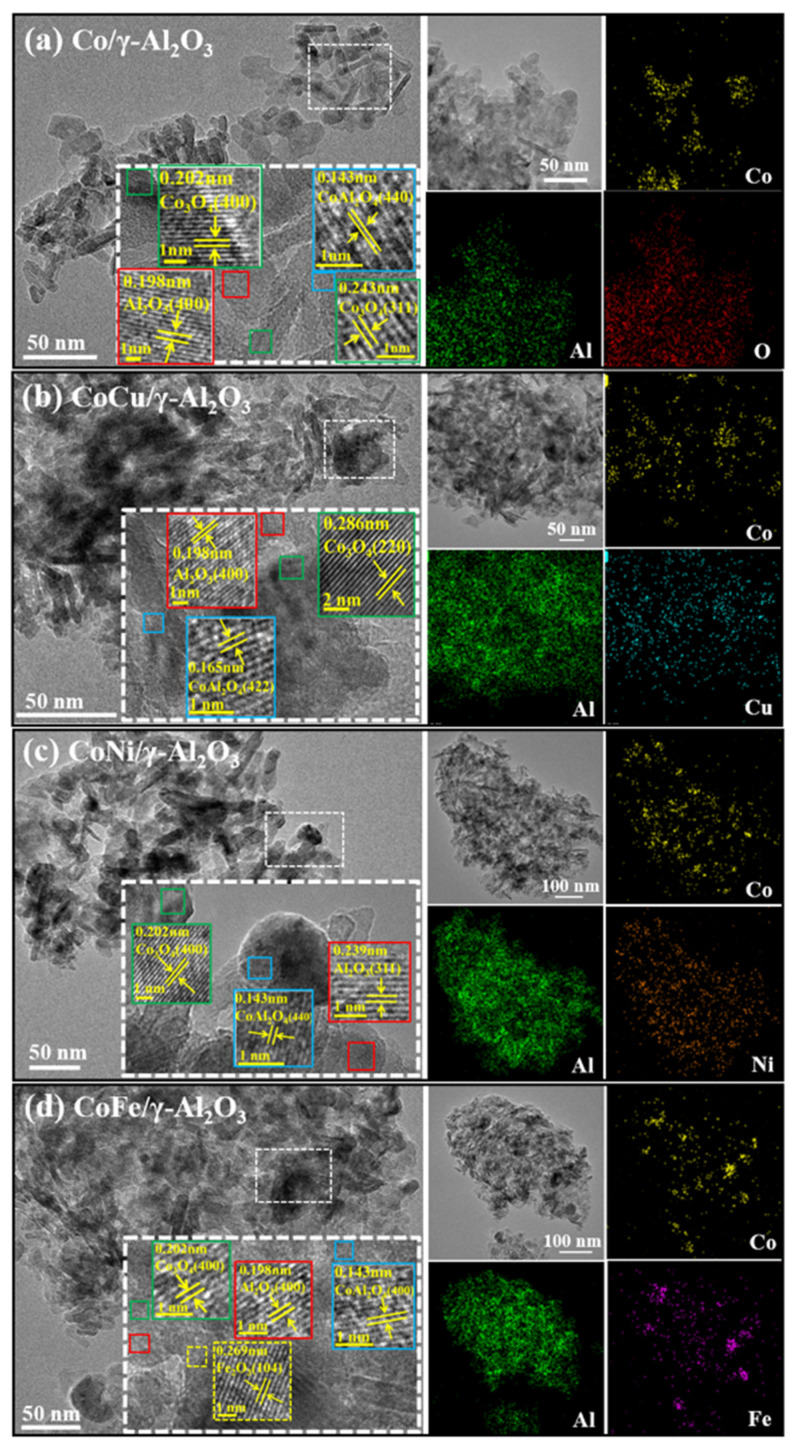
HRTEM and EDS mapping images of the fresh Co/γ-Al_2_O_3_ and CoM/γ-Al_2_O_3_ (M = Cu, Ni, or Fe) catalysts.

Figure 2 presents the HRTEM and EDS mapping images of the four alumina-supported metal catalysts. The catalysts exhibit an amorphous γ-Al_2_O_3_ structure, with no significant change in the morphology following the introduction of Cu, Ni, or Fe. Despite sulfate formation (as indicated by the S-O vibrational peaks at 1066 cm^−1^ in Figure S1) [37] and a slight increase in CoAl_2_O_4_ content (Figure S2) after sulfur poisoning, the overall catalyst structure remained stable, suggesting that the introduction of Cu, Ni, or Fe had no effect on the catalyst structure. Magnified images of fresh catalysts revealed that CoCu/γ-Al_2_O_3_, CoNi/γ-Al_2_O_3_, and CoFe/γ-Al_2_O_3_ present similar lattice fringes to Co/γ-Al_2_O_3_, corresponding to γ-Al_2_O_3_, Co_3_O_4_, and CoAl_2_O_4_ phases. Notably, only the CoFe/γ-Al_2_O_3_ catalyst showed the lattice fringe of Fe_2_O_3_ (104) [38], consistent with the XRD results. Additionally, EDS mapping analyses revealed the significant effect of the different metals on the dispersion of Co species on the support. Compared with that in Co/γ-Al_2_O_3_, Cu introduction enhanced the dispersion of Co species in CoCu/γ-Al_2_O_3_, while Ni and Fe introduction decreased it in CoNi/γ-Al_2_O_3_ and CoFe/γ-Al_2_O_3_, respectively. This effect was particularly pronounced in CoFe/γ-Al_2_O_3_, which exhibited agglomerated Co species. Concurrently, the Cu species were dispersed well in CoCu/γ-Al_2_O_3_, whereas the Fe species in CoFe/γ-Al_2_O_3_ were agglomerated significantly. The distinct dispersion behaviors of Co and M species in the CoM/γ-Al_2_O_3_ catalysts depend primarily on the interaction strength between Co and M. The formation of sulfates disrupts the dispersion of the original metal species, resulting in agglomeration. However, the strong interaction between Cu and Co is anticipated to maintain the effective dispersion of these metal species, even after sulfur poisoning, as illustrated in Figure S3. Conversely, the agglomeration of Co species in the CoNi/γ-Al_2_O_3_ catalyst became more pronounced after sulfur poisoning; this is primarily attributed to the weaker interaction between Ni and Co.

**Figure 3:**
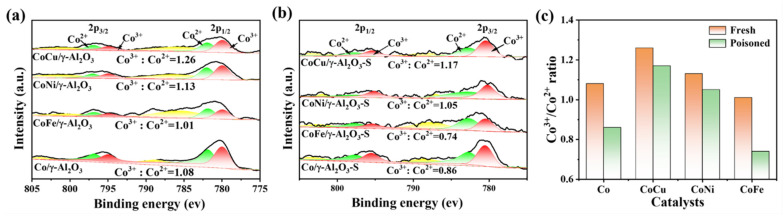
High-resolution Co 2p XPS profiles of (**a**) fresh and (**b**) sulfur-poisoned Co/γ-Al_2_O_3_ and CoM/γ-Al_2_O_3_ (M = Cu, Ni, or Fe) catalysts. (**c**) Co^3+^/Co^2+^ ratios of the different catalysts.

**Figure 4:**
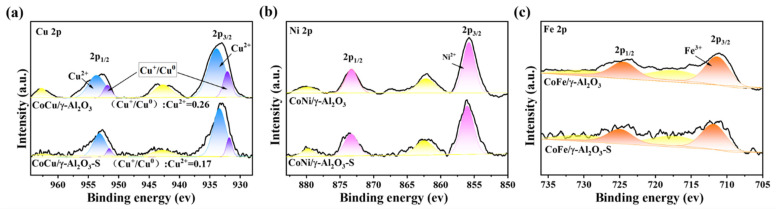
High-resolution XPS profiles of fresh and sulfur-poisoned CoM/γ-Al_2_O_3_ (M = Cu, Ni, or Fe) catalysts: (**a**) Cu 2p, (**b**) Ni 2p, and (**c**) Fe 2p.

We utilized XPS to initially investigate the valence states of Co species on the surface of each catalyst. As shown in Figure 3a, the CoCu/γ-Al_2_O_3_ catalyst exhibited the highest Co^3+^/Co^2+^ ratio of 1.26, whereas the CoNi/γ-Al_2_O_3_ and Co/γ-Al_2_O_3_ catalysts had comparable Co^3+^/Co^2+^ ratios of 1.13 and 1.08, respectively. The CoFe/γ-Al_2_O_3_ catalyst displayed the lowest ratio of 1.01. Following sulfur poisoning, the Co^3+^ content in all catalysts decreased, as shown in Figure 3b. This decrease is attributed to the formation of inactive CoSO_4_ through the reaction of active Co_3_O_4_ with sulfur species. The results in Figure 3c clearly reveal that the CoCu/γ-Al_2_O_3_ catalyst exhibited the smallest change in the active Co^3+^/Co^2+^ ratio after sulfur poisoning, exhibiting a decrease of only 0.09. This result suggests that the interaction between Cu and Co hinders the binding of Co^3+^ to sulfur species. Additionally, we analyzed the valence states of the doped metals: Cu, Ni, and Fe. For the CoCu/γ-Al_2_O_3_ catalyst, an XPS peak corresponding to Cu^2+^ species was observed at 933.9 eV, along with a minor peak attributed to low-valence Cu^+^ (Cu^0^) species at 932.1 eV (Figure 4a). The peaks at 571 and 569 eV in the Cu LMM Auger spectrum (see Figure S4) also confirmed the coexistence of Cu^+^ and Cu^0^ species [39]. Despite the reaction of some CuO_x_ with sulfur species to form CuSO_4_ upon sulfur poisoning, the surface of CoCu/γ-Al_2_O_3_ maintained significant amounts of Cu^+^ and Cu^0^. Conversely, the Ni 2p and Fe 2p XPS profiles (Figure 4b,c) indicate a single oxidation state for NiO and Fe_2_O_3_ in CoNi/γ-Al_2_O_3_ and CoFe/γ-Al_2_O_3_, respectively [40,41,42]. It is well known that transition metals with variable oxidation states are beneficial for promoting the redox performance of catalysts. The CoCu/γ-Al_2_O_3_ catalyst contains Cu^2+^/Cu^+^(Cu^0^) and Co^3+^/Co^2+^ redox pairs, which can facilitate electron transfer and promote the redox reaction of Cu^+^(Cu^0^) + Co^3+^ ↔ Cu^2+^ + Co^2+^. Consequently, Cu and Co exhibit a strong synergistic effect, with Cu inhibiting the formation of inactive CoAl_2_O_4_ from Co_3_O_4_ and enhancing the dispersion of Co species on the support, thereby maintaining the stable, active Co_3_O_4_ phase. At the same time, Co also promotes electron transfer between different Cu oxide species with different valence states, enabling the formation of highly active Cu^+^ (Cu^0^) species, thereby enhancing the catalytic redox performance. In contrast, the weaker interaction between Ni and Co in the CoNi/γ-Al_2_O_3_ catalyst has a minimal effect on the state of Co species. The greater decrease in the Co^3+^/Co^2+^ ratio of the CoFe/γ-Al_2_O_3_ catalyst after poisoning is due to the inability of Fe to influence the dispersion state of Co, causing it to further aggregate and more easily react with sulfur species to form inactive CoAl_2_O_4_.

**Figure 5:**
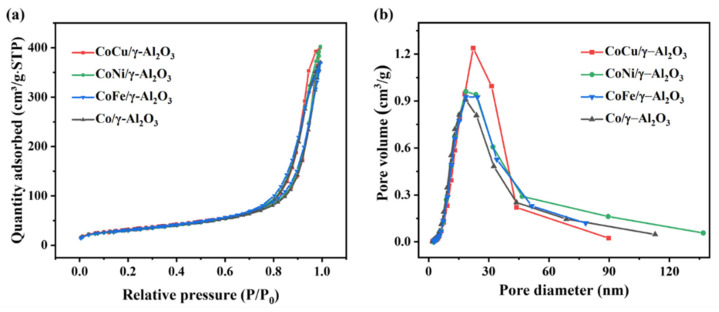
N_2_ adsorption/desorption isotherms and (**b**) pore-size distributions (**a**) of the fresh Co/γ-Al_2_O_3_ and CoM/γ-Al_2_O_3_ (M = Cu, Ni, or Fe) catalysts.

Figure 5a presents the N_2_ adsorption/desorption isotherms of the four fresh catalysts. According to the IUPAC classification, all samples presented Type IV isotherms characterized by a H_3_ hysteresis loop, indicating that they maintained a consistent slit-like mesoporous structure [21]. Figure 5b reveals a narrow pore-size distribution, with the pore sizes ranging from 6 to 45 nm. Notably, among the different catalysts, CoCu/γ-Al_2_O_3_ had superior pore-size uniformity and the largest *S*_BET_ (Table 1). Furthermore, owing to the similar radii of Cu^2+^ (0.73 Å) and Co^2+^ (0.79 Å) [43], the introduction of Cu enhances the pore volume (*V*_p_), enabling the metal ions to form composite oxides with the support, thus promoting pore-size uniformity. Upon sulfur poisoning, the CoNi/γ-Al_2_O_3_ and CoFe/γ-Al_2_O_3_ catalysts experience sulfate accumulation, resulting in a decrease in both *S*_BET_ and *V*_p_. Specifically, in the CoFe/γ-Al_2_O_3_ catalyst, the agglomeration of Fe and Co species exacerbates pore blockage, leading to a notable decrease in the pore volume.

### 2.2. The Effect of the Additional Metal on the Acidity of the Catalyst

The surface acidity and basicity of multi-metal oxide catalysts are critical factors that influence their catalytic efficiency. During the catalysis of VOC oxidation, acidic sites on the support enhance the adsorption and activation of the VOCs on the active sites of the catalyst surface, accelerating their catalytic degradation [44].

**Figure 6:**
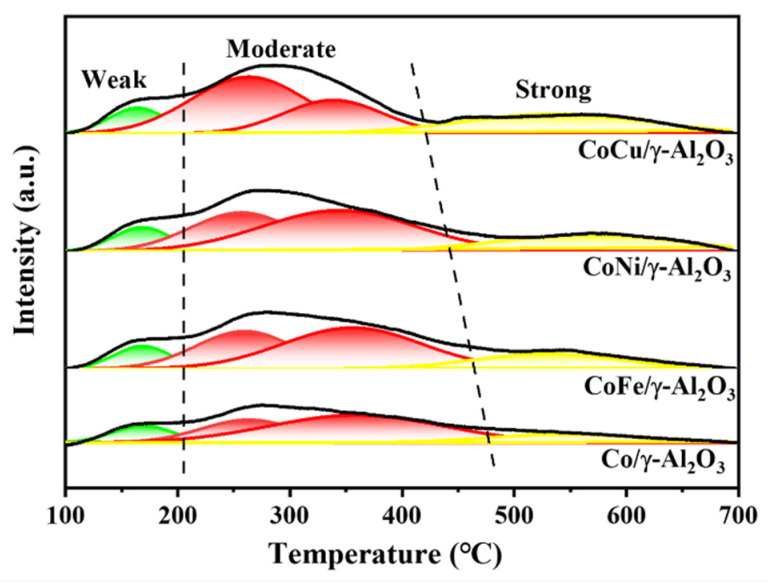
NH_3_-TPD curves of the fresh Co/γ-Al_2_O_3_ and CoM/γ-Al_2_O_3_(M = Cu, Ni, or Fe) catalysts.

We comprehensively analyzed the influence of adding a second metal on the acidity of the Co/γ-Al_2_O_3_ catalyst by using NH_3_-TPD experiments (Figure 6). The Co/γ-Al_2_O_3_ catalyst contains numerous weak-to-moderately strong acidic sites, as well as a limited number of strong acidic sites. After the introduction of Cu, Ni, or Fe, the number of acidic sites on the catalyst differed. Specifically, the CoCu/γ-Al_2_O_3_ catalyst exhibited a significant increase in moderately strong and strong acidic sites (0.10390 mmol NH_3_/g_cat._), and the CoNi/γ-Al_2_O_3_ catalysts also developed a specific level of strong acidic sites (0.08208 mmol NH_3_/g_cat._). In contrast, the acidity of the CoFe/γ-Al_2_O_3_ catalyst increased only slightly, and the detailed data are presented in Table 2. The addition of a second metal improves the acidity of the Co/γ-Al_2_O_3_ catalyst, likely due to the interactions between the added metal, Co, and the γ-Al_2_O_3_ support. A stronger interaction between the metal species enhances the ability of the acidic sites to bind protons within the catalytic system, resulting in an overall increase in the acidity of the system.

**Figure 7:**
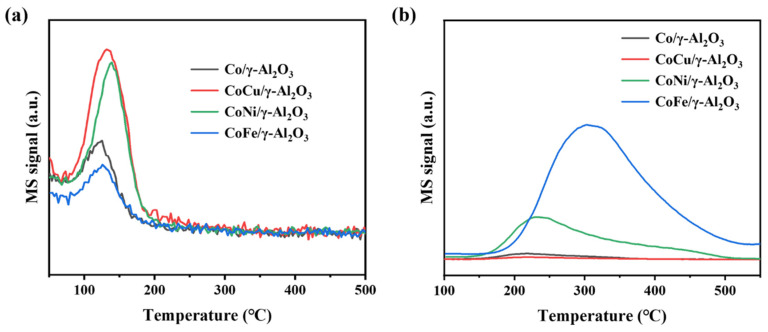
Toluene-TPD (**a**) and SO_2_-TPD (**b**) results for the fresh Co/γ-Al_2_O_3_ and CoM/γ-Al_2_O_3_ (M = Cu, Ni, or Fe) catalysts.

Toluene-TPD tests confirmed that increasing the strongly acidic sites favored toluene adsorption (Figure 7a). The toluene desorption peak areas of the CoCu/γ-Al_2_O_3_ and CoNi/γ-Al_2_O_3_ catalysts were notably larger than those of CoFe/γ-Al_2_O_3_ and Co/γ-Al_2_O_3_, suggesting higher abundance of toluene adsorption sites on their surfaces. Catalysts with a higher concentration of strongly acidic sites exhibit enhanced toluene adsorption, which facilitates the activation of C-C and C-H bonds [45]. Conversely, the CoFe/γ-Al_2_O_3_ catalyst with weaker acidity demonstrated the lowest toluene adsorption capacity. Furthermore, the characteristics of the acidic centers in a catalyst influence its SO_2_ adsorption capacity. In SO_2_-TPD experiments, the CoFe/γ-Al_2_O_3_ catalyst provided the largest SO_2_ adsorption peak area and higher adsorption temperatures (Figure 7b), suggesting a strong adsorption of SO_2_, with a lower likelihood of the desorption of the adsorbed sulfur species, indicating increased risk of sulfur poisoning. The CoNi/γ-Al_2_O_3_ catalyst exhibited specific adsorption capacity for SO_2_. In contrast, the CoCu/γ-Al_2_O_3_ and Co/γ-Al_2_O_3_ catalysts exhibited limited SO_2_ adsorption capacity, indicating that in sulfur-rich environments, only a small fraction of the surface species interact with sulfur species and a larger proportion of the active components can be retained. Consequently, they are more suitable for the catalytic oxidation of sulfur-containing VOCs.

### 2.3. Evaluation of Catalytic Activity and Sulfur Tolerance

**Figure 8:**
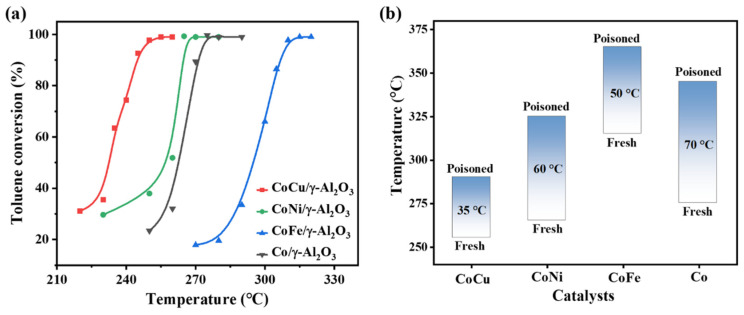
(**a**) Toluene conversion on fresh Co/γ-Al_2_O_3_ and CoM/γ-Al_2_O_3_ (M = Cu, Ni, or Fe) catalysts. (**b**) Comparison of T_99_ for toluene combustion over fresh and sulfur-poisoned Co/γ-Al_2_O_3_ and CoM/γ-Al_2_O_3_ (M = Cu, Ni, or Fe) catalysts.

Figure 8a displays the catalytic activities of the single-metal Co/γ-Al_2_O_3_ catalyst and the three CoM/γ-Al_2_O_3_ systems (M = Cu, Ni, or Fe) in toluene combustion. The introduction of Cu, Ni, and Fe notably affected the catalytic activity of the Co/γ-Al_2_O_3_ system. Among the catalysts, CoCu/γ-Al_2_O_3_ demonstrated the strongest performance, reducing the temperature required for the complete conversion of toluene (T_99_) by 20 °C (from 275 °C for Co/γ-Al_2_O_3_ to 255 °C for CoCu/γ-Al_2_O_3_). The CoNi/γ-Al_2_O_3_ catalyst decreased the T_99_ of toluene by 10 °C (to 265 °C). In sharp contrast, the CoFe/γ-Al_2_O_3_ catalyst increased it by 40 °C (to 315 °C). The superior performance of the CoCu/γ-Al_2_O_3_ catalyst is due to the synergistic effect of Cu and Co, which prevents the formation of inactive CoAl_2_O_4_ by the reaction of active Co_3_O_4_ with the γ-Al_2_O_3_ support, maintaining more active species. This synergistic effect also facilitates the formation of high-performance Cu^+^(Cu^0^) species, resulting in two catalytic redox pairs (Cu^2+^/Cu^+^(Cu^0^) and Co^3+^/Co^2+^) in the system. Furthermore, the cumulative interactions among Cu, Co, and γ-Al_2_O_3_ strengthen the acidity of the system, improving toluene adsorption and activation, and enhancing the catalyst efficiency. Despite the weak interaction between Ni and Co in the CoNi/γ-Al_2_O_3_ catalyst, the increased number of strongly acidic sites further enhances the adsorption and activation of toluene, thereby moderately improving its catalytic activity in toluene combustion. Conversely, aggregated Fe_2_O_3_ in the CoFe/γ-Al_2_O_3_ catalyst weakens the stability of Co_3_O_4_, promoting its combination with γ-Al_2_O_3_ to form inactive CoAl_2_O_4_, and blocks the acidic sites. This reduces toluene adsorption and activation, thereby significantly increasing the toluene combustion temperature.

We comprehensively analyzed the sulfur resistance of the CoM/γ-Al_2_O_3_ catalysts with Cu, Ni, and Fe (Figure S5). A comparison of the catalytic activities of the four systems before and after sulfur poisoning (Figure 8b) revealed that the sulfur species has an adverse effect on the toluene combustion performance of the catalysts. This is mainly due to the formation of stable sulfates through reactions between the catalytically active sites and sulfur species. However, the introduction of Cu, Ni, and Fe improved the sulfur resistance of the Co/γ-Al_2_O_3_ system to some extent. After the introduction of sulfur, the T_99_ for toluene degradation on the Co/γ-Al_2_O_3_ system increased by 70 °C (from 275 °C before sulfur poisoning to 345 °C after sulfur poisoning). The T_99_ of the CoCu/γ-Al_2_O_3_ catalyst increased by only 35 °C (from 255 to 290 °C), whereas the T_99_ values of the CoNi/γ-Al_2_O_3_ and CoFe/γ-Al_2_O_3_ catalysts increased more significantly, by 60 °C (from 265 to 325 °C) and 50 °C (from 315 to 365 °C), respectively. Additionally, the CoCu/γ-Al_2_O_3_ catalyst exhibited remarkable stability, maintaining consistent toluene conversion above 82% throughout the entire 72 h continuous operation. The strong synergistic effect between Cu and Co in the CoCu/γ-Al_2_O_3_ catalyst, coupled with the presence of Cu^2+^/Cu^+^(Cu^0^) and Co^3+^/Co^2+^ redox pairs, enhanced the stability and redox capacity of the system. This allows the active species to remain stable in the presence of sulfur, moderately inhibiting their combination with sulfur species and leading to only a slight activity decrease after sulfur poisoning. The weaker interactions between Co and Ni or Fe following sulfur introduction not only destabilize the active cobalt species but also allow the active species to further combine with sulfur, resulting in the formation of sulfates and significantly decreased catalytic activity after sulfur poisoning.

## 3. Experimental Methods

### 3.1. Catalyst Preparation

The CoM/γ-Al_2_O_3_ (M = Cu, Fe, or Ni) catalysts were prepared by using an impregnation method. γ-Al_2_O_3_ (99.99%, 110–150 m^2^/g, 40–60 mesh, amorphous particles, 3A, LOT# 07CXRJNE, Anqing, China) was impregnated with mixed metal solutions composed of cobalt nitrate (Co(NO_3_)_2_∙6H_2_O, 99.99%, Energy Chemical, Shanghai, China) and another metal nitrate (Cu(NO_3_)_2_∙3H_2_O, 99%, 3A, Shanghai, China), (Fe(NO_3_)_3_∙9H_2_O, 99%, Energy Chemical, Shanghai, China) or (Ni(NO_3_)_2_∙6H_2_O, 98%, DAMAO, Tianjin, China) in 1:1 molar ratios. The metal-impregnated supports were then dried and calcined at 500 °C for 2 h to obtain three 10% CoM/γ-Al_2_O_3_, viz., CoCu/γ-Al_2_O_3_, CoNi/γ-Al_2_O_3_, and CoFe/γ-Al_2_O_3_. A 10% Co/γ-Al_2_O_3_ catalyst, composed solely of cobalt, was synthesized following a similar procedure for use as a reference.

Sulfur-poisoned catalysts were prepared through an in situ thermal decomposition process using ammonium sulfate as the sulfur source, achieving rapid and quantitative sulfur incorporation. The catalysts were immersed in a 0.1 mol/L (NH_4_)_2_SO_4_ solution for 10 h and then calcined at 500 °C for 2 h to obtain sulfur-poisoned catalysts with a sulfur content of 0.4 wt.%. The sulfur-poisoned catalysts are denoted as CoCu/γ-Al_2_O_3_-S, CoNi/γ-Al_2_O_3_-S, CoFe/γ-Al_2_O_3_-S, and Co/γ-Al_2_O_3_-S.

### 3.2. Catalytic Performance Evaluation

The catalytic activities of the catalysts were assessed, before and after sulfur poisoning, by using an FD-2000 fixed-bed reactor (Huasi, Changsha, China) at a gas hourly space velocity of 20,000 h^−1^ and a toluene concentration of 1000 ppm. The toluene concentration was monitored by using an online gas chromatograph (GC-1690, Jiedao, Hangzhou, China) equipped with a flame ionization detector.

The VOC concentration was adjusted to approximately 1000 ppm by using an external standard method. Toluene (0.5 μL) was taken in a sealed 100 mL syringe and heated under an infrared lamp to vaporize it, thereby obtaining a calibration gas with a toluene concentration of 0.88 × 10^−6^ g/mL (0.79 × 10^−6^, 0.87 × 10^−6^ g/mL). Then, 0.2, 0.4, 0.6, 0.8, and 1.0 mL of the calibration gas were used for gas chromatography to construct a calibration curve.

### 3.3. Catalyst Characterization

The XRD patterns of the catalysts were recorded by using a Rigaku X-Ray diffractometer (SmartLab 9KW, Rigaku, Tokyo, Japan). The FT-IR spectra were recorded on a Fourier transform infrared spectrometer (Nicolet 5700, Thermo Scientific, Waltham, MA, USA). To determine the specific surface area (*S*_BET_) and pore-size distribution of the catalyst, N_2_ adsorption/desorption experiments were conducted by using an ASAP-2460 multistation analyzer (Micromeritics, Norcross, GA, USA). HRTEM (FEI Talos F200x, FEI, Hillsboro, OR, USA) and EDS (SuperX, Thermo Scientific, USA) were performed to obtain morphological and elemental information. XPS analysis was conducted on an Escalab Xi+ instrument (Thermo Fisher Scientific, Waltham, MA, USA) using Al *Kα* radiation (1486.6 eV); the XPS peaks were calibrated with respect to the C 1s peak at 284.8 eV. NH_3_-TPD and H_2_-TPR were conducted by using a fully automated chemical sorption analyzer (Autochem II, Micromeritics, USA). For Toluene-TPD and SO_2_-TPD, a quadrupole gas analyzer (QGA; Hiden, Warrington, UK) was employed to monitor toluene and SO_2_ concentrations in real time.

## 4. Conclusions

We successfully synthesized a series of alumina-supported bimetallic oxide catalysts, CoM/γ-Al_2_O_3_ (M = Cu, Ni, or Fe), by using an impregnation protocol and investigated the synergistic effects of the metal species in the catalytic combustion of toluene. The introduction of Cu significantly enhanced the activity of the Co/γ-Al_2_O_3_ catalyst, decreasing the temperature for the complete conversion of toluene by 20 °C, while the addition of Fe had a detrimental effect on the performance of Co/γ-Al_2_O_3_. The synergistic interaction between Cu and Co effectively suppressed the formation of inactive CoAl_2_O_4_, maintaining a higher concentration of active Co_3_O_4_ species and simultaneously improving the redox capacity of the system. Cu incorporation also increased the acidity of the catalyst, optimizing the adsorption and activation of toluene, thus enhancing the catalytic efficiency. Under sulfur-poisoning conditions, Cu introduction significantly improved the sulfur resistance of the catalyst, ensuring that the mixed-metal CoCu/γ-Al_2_O_3_ catalyst maintained a high activity in the presence of sulfur. This research provides a strong theoretical foundation for the development of high-performance multi-metallic catalytic systems and their application in complex environments containing H_2_O or CO_2_.

## Figures and Tables

**Table 1:** Textural properties of the fresh Co/γ-Al_2_O_3_ and CoM/γ-Al_2_O_3_ catalysts (M = Cu, Ni, or Fe).

Sample	Co/γ-Al_2_O_3_		CoCu/γ-Al_2_O_3_		CoNi/γ-Al_2_O_3_		CoFe/γ-Al_2_O_3_	
	Fresh	Poisoned	Fresh	Poisoned	Fresh	Poisoned	Fresh	Poisoned
***S*_BET_ ^a^ (m^2^/g)**	112.4	100.6	117.6	121.0	111.9	114.2	110.8	102.7
***V*_p_ ^b^ (m^3^/g)**	0.5740	0.5614	0.6205	0.6591	0.6200	0.5872	0.5706	0.5339

^a^ BET specific surface area. ^b^ Total pore volume estimated at P/P_0_ = 0.99.

**Table 2:** NH_3_ consumption of the fresh Co/γ-Al_2_O_3_ and CoM/γ-Al_2_O_3_(M = Cu, Ni, or Fe) catalysts based on NH_3_-TPD results.

Sample	Weak (mmol/g_cat._)	Moderate (mmol/g_cat._)	Strong (mmol/g_cat._)
CoCu/γ-Al_2_O_3_	0.04248	0.28546	0.10390
CoNi/γ-Al_2_O_3_	0.03998	0.29940	0.08208
CoFe/γ-Al_2_O_3_	0.03992	0.29330	0.06744
Co/γ-Al_2_O_3_	0.03986	0.27139	0.04998

## Data Availability

Data are contained within the article.

## Supplementary Materials

The following supporting information can be downloaded at: https://www.mdpi.com/article/10.3390/molecules30051188/s1, Figure S1: (a) FT-IR spectra and (b) High-resolution S 2p XPS profiles of sulfur-poisoned Co/γ-Al_2_O_3_ and CoM/γ-Al_2_O_3_ (M = Cu, Ni, or Fe) catalysts. Figure S2: XRD patterns of sulfur-poisoned Co/γ-Al_2_O_3_ and CoM/γ-Al_2_O_3_ (M = Cu, Ni, or Fe) catalysts. Figure S3: TEM and EDS mapping images of sulfur-poisoned Co/γ-Al_2_O_3_ and CoM/γ-Al_2_O_3_ (M = Cu, Ni, or Fe) catalysts. Figure S4: High-resolution Cu LMM Auger spectra of the CoCu/γ-Al_2_O_3_ catalyst before and after sulfur poisoning. Figure S5: Toluene conversion over sulfur-poisoned Co/γ-Al_2_O_3_ and CoM/γ-Al_2_O_3_ (M = Cu, Ni, or Fe) catalysts. Figure S6: Stability testing of CoCu/γ-Al_2_O_3_ catalyst for toluene combustion.
