# Supplementary material for: Comprehensive Analysis of the Synergistic Effects of Bimetallic Oxides in CoM/γ-Al2O3 (M = Cu, Fe, or Ni) Catalysts for Enhancing Toluene Combustion Efficiency
Source: Molecules. 2025 Mar 6;30(5):1188. doi: 10.3390/molecules30051188 (PMC11901707; doi:10.3390/molecules30051188)
Supplement: Supplementary file 1 [file molecules-30-01188-s001.zip › molecules-3489658-supplementary.pdf]

Comprehensive Analysis of the Synergistic Effects of Bimetallic Oxides in CoM/γ-Al_2O_3 (M = Cu, Fe, or Ni) Catalysts for Enhancing Toluene Combustion Efficiency

Yuwei Tang [1], Xu Yang [1], Qinglong Zhang [1], Dongmei Lv [1], Shufeng Zuo [2,*] and Jing Li [1,*]

[1] Shandong Provincial Key Laboratory of Chemical Energy Storage and Novel Cell Technology, College of Chemistry and Chemical Engineering, Liaocheng University, Liaocheng 252059, China; tangyuweiyibo@163.com (Y.T.); yxv2021@163.com (X.Y.); a1942213564@163.com (Q.Z.); lvdongmei@lcu.edu.cn (D.L.)

[2] Zhejiang Key Laboratory of Alternative Technologies for Fine Chemicals Process, College of Chemistry and Chemical Engineering, Shaoxing University, Shaoxing 312000, China

* Correspondence: sfzuo@usx.edu.cn (S.Z.); muzianty@126.com (J.L.)

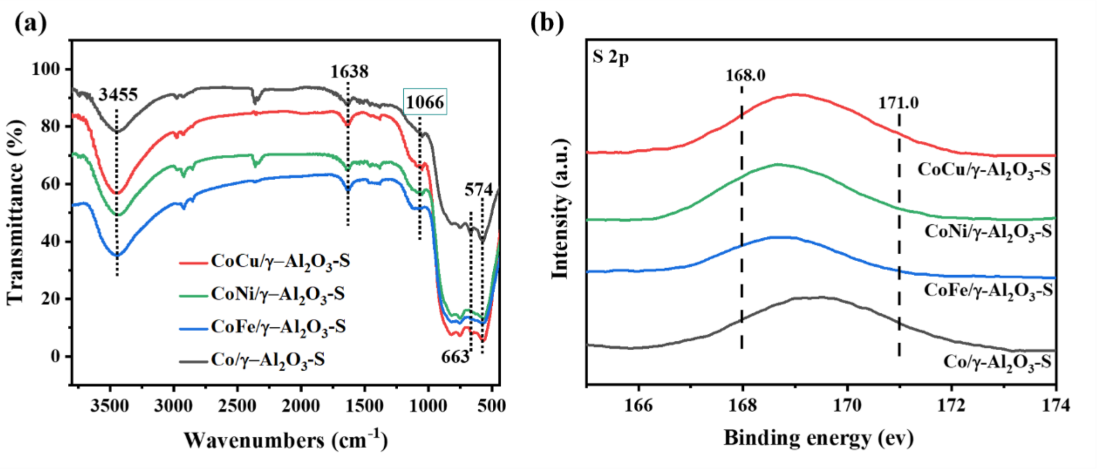

Figure S1. (**a**) FT-IR spectra and (**b**) High-resolution S 2p XPS profiles of sulfur-poisoned Co/γ-Al_2O_3 and CoM/γ-Al_2O_3 (M = Cu, Ni, or Fe) catalysts.

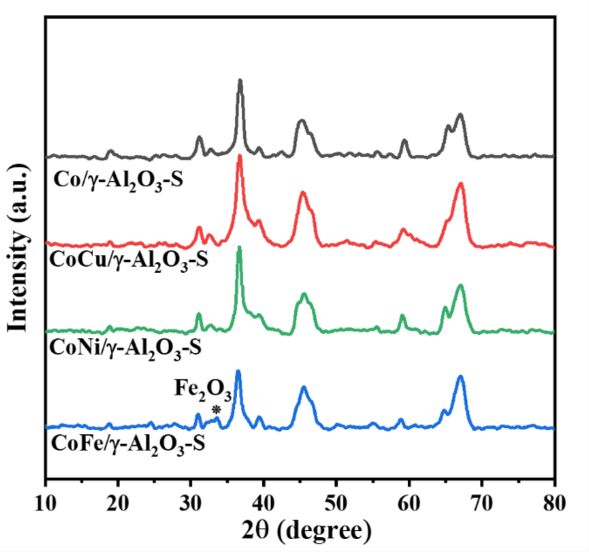

Figure S2. XRD patterns of sulfur-poisoned Co/γ-Al_2O_3 and CoM/γ-Al_2O_3 (M = Cu, Ni, or Fe) catalysts. (* used to mark the characteristic of Fe_2O_3)

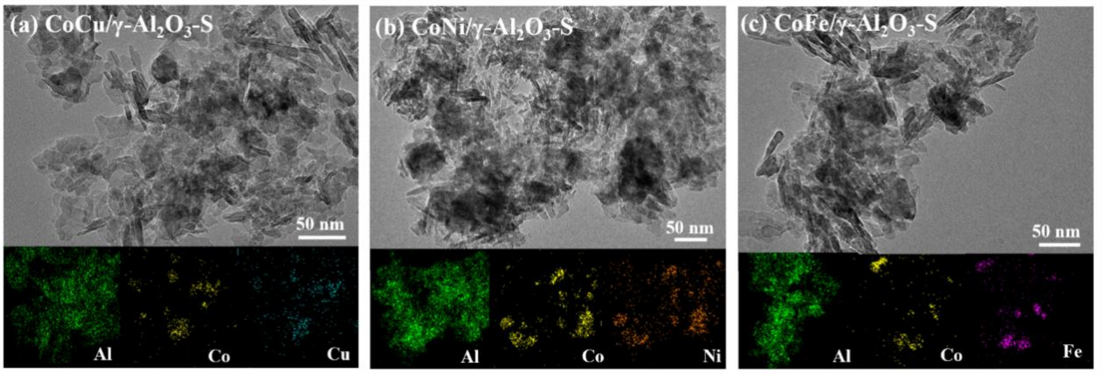

Figure S3. TEM and EDS mapping images of sulfur-poisoned Co/γ-Al_2O_3 and CoM/γ-Al_2O_3 (M = Cu, Ni, or Fe) catalysts.

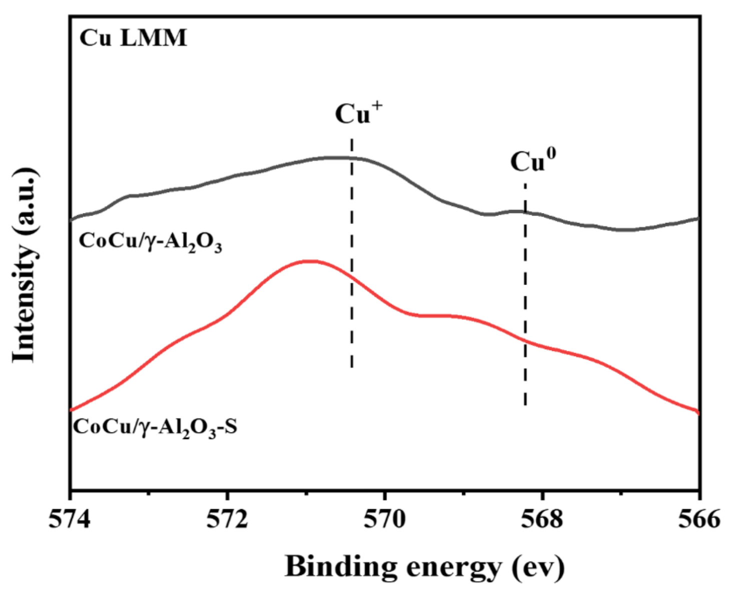

Figure S4. High-resolution Cu LMM Auger spectra of the CoCu/γ-Al_2O_3 catalyst before and after sulfur poisoning.

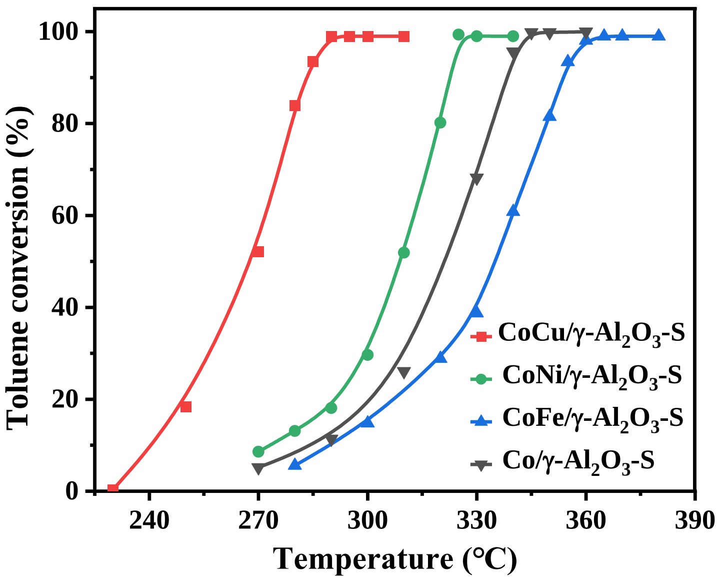

Figure S5. Toluene conversion over sulfur-poisoned Co/γ-Al_2O_3 and CoM/γ-Al_2O_3 (M = Cu, Ni, or Fe) catalysts.

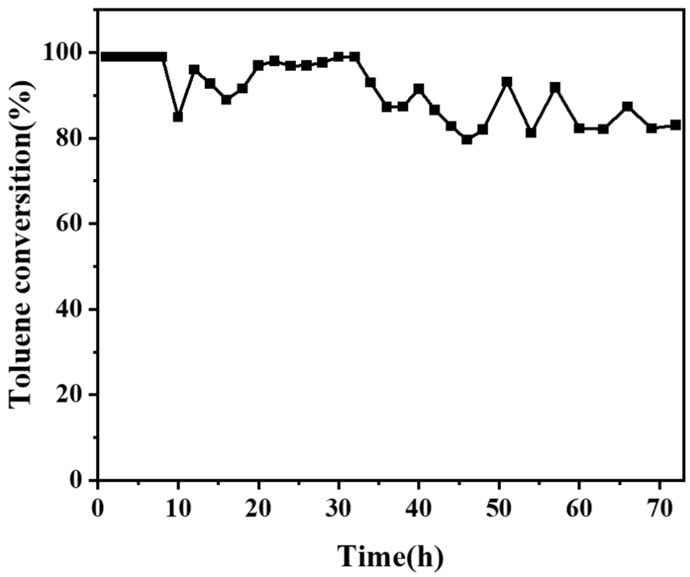

Figure S6. Stability testing of CoCu/γ-Al_2O_3 catalyst for toluene combustion.
